# Antidiabetic and *In Vitro* Enzyme Inhibition Studies of Methanol Extract of *Ocimum tenuiflorum* Linn Leaves and Its Fractions

**DOI:** 10.21315/tlsr2020.31.1.9

**Published:** 2020-04-07

**Authors:** Leila Mousavi, Rabeta Mohd Salleh, Vikneswaran Murugaiyah

**Affiliations:** 1 Food Technology Division, School of Industrial Technology, Universiti Sains Malaysia, 11800 USM Pulau Pinang, Malaysia; 2 Discipline of Pharmacology, School of Pharmaceutical Sciences, Universiti Sains Malaysia, 11800 USM Pulau Pinang, Malaysia

**Keywords:** α-amylase, α-glucosidase, *In vitro*, Insulin level, *Ocimum tenuiflorum* L, α-amilase, α-glukosidase, *In vitro*, Aras insulin, *Ocimum tenuiflorum* L

## Abstract

The current study aimed to determine the best dose of methanol extract of *Ocimum tenuiflorum* L. leaves extract, and it is a fraction to blood-glucose-lowering in diabetic rats, and evaluated the α-amylase, α-glucosidase inhibitors and insulin level of diabetic rats used to achieve greater control over hyperglycemia. The result of the antihyperglycaemic of oral administration of a different dose of methanol extract in streptozotocin-induced rats showed that the highest dose of methanol extract significantly reduced the blood glucose level compared to another dose. Also, the result of repeated administration of methanol fractions indicates that ethyl acetate-butanol fraction exhibited a stronger antihyperglycemic effect than chloroform and ethanol-water fractions. Moreover, the result showed that effect of methanol extract and its fraction on α-glucosidase and α-amylase enzymes activities and its insulin level by in vitro study, ethyl acetate-butanol fraction could control with low concentration compared to other fractions and acarbose that used as a positive control. From the result of insulin level, methanol extract and fraction did not show any significant. These findings indicated that the active crude extract (methanol) and its active fractions (ethyl acetate/butanol) could exert significant glucose-lowering effect due to the presence of polyphenolics active constituents. In conclusion, isolation of the active components of *Ocimum tenuiflorum* L. may pave the way to the development of new agents for the treatment of diabetes and its complications.

## Abstract (ms)

Tujuan kajian ini adalah untuk menentukan dos terbaik ekstrak metanol daun *Ocimum tenuiflorum* dan fraksinya dalam menurunkan glukosa darah tikus yang diabetik, dan menilai perencat α-amilase, α-glukosidase dan aras insulin tikus diabetik yang digunakan dalam mencapai pengawalan hiperglisemia yang lebih besar. Keputusan pemberian oral anti hiperglisemia dos berbeza ekstrak metanol pada tikus teraruh streptozotosin menunjukkan dos ekstrak metanol yang paling tinggi merendahkan aras glukosa darah secara signifikan berbanding dengan dos yang lain. Tambahan pula, keputusan pemberian fraksi metanol secara berulang menunjukkan fraksi etil asetat-butanol mempamerkan kesan antihiperglisemia lebih kuat berbanding fraksi klorofom dan etanol-air. Lebih-lebih lagi, keputusan menunjukkan kesan ekstrak metanol dan fraksinya pada aktiviti enzim α-amilase dan α-glukosidase dan aras insulinnya secara kajian *in vitro,* fraksi etil asetat-butanol dapat mengawal dengan kepekatan rendah berbanding dengan fraksi lain dan akarbos yang digunakan sebagai kawalan positif. Daripada keputusan aras insulin, ekstrak metanol dan fraksi tidak menunjukkan apa-apa signifikan. Penemuan Ini menunjukkan ekstrak kasar yang aktif (metanol) dan fraksi aktif (etil asetat/butanol) berupaya memberi kesan penurunan glukosa disebakan kehadiran bahan aktif polifenolik. Kesimpulannya, isolasi komponen aktif *Ocimum tenuiflorum* L. dapat memberi laluan pada pembangunan agen baharu bagi rawatan diabetik dan komplikasinya.

**Highlights**

- The highest dose of methanol extract significantly reduced the blood glucose level compared to another dose.
- The ethyl acetate-butanol fraction exhibited a stronger antihyperglycemic effect compared to chloroform and ethanolwater fractions.
- The ethyl acetate-butanol fraction was identified as a best fraction to reduce a α-glucosidase and α-amylase enzymes activities and insulin level with low concentration compared to other fractions and acarbose.

## INTRODUCTION

In 2016, there were 1.6 million diabetes deaths in the world (World Health Organisation 2016) and over 3,492,600 cases of diabetes reported in Malaysia (International Diabetes Federation 2017) last year.

Despite the availability of various types of antidiabetic drugs, it is necessary to search for new therapeutic agents because of the side effects associated with existing treatments and the diminished response that comes with their prolonged use (Kothari *et al.* 2004; Mukherjee *et al.* 2006). Balamurugan *et al.* (2014) identified over 400 plant species with hypoglycaemic properties, and Lee *et al.* (2003) reported on the effects of many of these species on the activity of liver hexokinase. Furthermore, Khan *et al.* (2010) and Kumar *et al.* (2011) reported that treatment with medicinal herbs had improved the activity of liver glucose-6-phosphatase, glycogen synthase, glycogen phosphorylase, glucose-6-phosphate dehydrogenase, and phosphor-fructokinase. Other herbal crude extracts may be useful for restoring the activity of serum enzymes, such as alkaline phosphatase (ALP), acid phosphatase, lactate dehydrogenase (LDH) and transaminases (Eskander *et al.* 1995).

*Ocimum tenuiflorum* L. belongs to the Lamiaceae family. In Malaysia, *Ocimum tenuiflorum* L. is known as *ruku*, and many people use the fresh *Ocimum tenuiflorum* L. young leaves as a regular part of their daily diet to make *nasi ulam* (i.e., mixed herb rice). *Ocimum tenuiflorum* L. is suggested by ethnobotanical reports for its some medicinal properties and act as an antidiabetic, anti-fertility, hypotensive, hypolipidemic, anticancer, anti-asthmatic, antiemetic, diaphoretic and anti-stress agent (Kothari *et al.* 2004). The leaf or entire plant is used in Thailand to treat vomiting (Mukherjee *et al.* 2006).

On the other hand, the different organic crude extracts from the *Ocimum tenuiflorum* L. leaves and stem display antimicrobial activity against some strains of bacteria, fungi and yeast species (Mousavi *et al*. 2014).

In the present study, the fractionation of methanol extract aims to determine which fraction is more potent hence pave the way for chemical characterisation and standardisation. This finding will indicate which compounds are responsible for antidiabetic activity. Besides, the importance of the *Ocimum tenuiflorum* L. plant in treatment of diabetes, there are no previous reports that studied in detail (*in vitro*) the effects of the *Ocimum tenuiflorum* L. extract and its fractions on α-glucosidase and α-amylase inhibitors and insulin level. Thus, the present study investigated extract and fractions of *Ocimum tenuiflorum* L. for *in vitro* against α-glucosidase, α-amylase inhibitory activity, and effect in insulin level.

## MATERIALS AND METHODS

### Plant Material and Preparation of Sample

The fresh sample of *Ocimum tenuiflorum* L. (Lamiaceae) was collected from Perak and identified and kept at the Herbarium of the School of Biological Sciences, Universiti Sains Malaysia (USM Herbarium number 11400). Followed that flowers and leaves removed from the stems and tap water was used for washing and rinsed with distilled water. Leaves and stems were frozen for three days separately (Millrok Technology, LD53, Kingston, USA) (Ratti 2001). Then, a dried sample was used for the blending (Panasonic, MX 335, Malaysia) and was kept in vacuum package at 4°C (Toshiba, GR-M48MP, Minato-Ku, Japan) before the analysis process.

### Extraction Procedure

The extraction procedure was conducted by maceration method (40°C–60°C) with about 200 g of sample with five solvents, i.e., n-hexane (HE), chloroform (CE), ethyl acetate (EE), methanol (ME), water (WE) for seven consecutive days. The extracts obtained were filtered with Whatman paper No. 1 and concentrated in vacuum using rotary evaporation (Buchi, Switzerland, EYELA N1200B 1101348) at reduced pressure and 30°C. The concentrated extracts (HE, CE, EE, ME, WE) were dried in the oven (40°C) until the organic solvent evaporated, while the concentrated WE were dried in a freeze dryer. The dried extracts were kept in the freezer (−25°C) until further analysis. All extracts first were dissolved using 5% Tween 80 in normal saline before use.

### Preparation of Fractions

Fractionation was conducted by 25 g of the most active extract (ME) by liquid-liquid extraction method with five solvents, i.e., chloroform, ethyl acetate, n-butanol, ethanol and water using separating funnel consequently and then concentrated in vacuum by rotary evaporation (Buchi, Switzerland, EYELA N1200B 1101348) at reduced pressure and 30°C–40°C. The concentrated fractions (CF, EAF, EF, nBF, WF) were dried in an oven (40°C) until organic solvent evaporated, whereas the concentrated WF dried in the freeze dryer and kept in the freezer (−25°C) till further analysis. All fractions were dissolved in 5% Tween 80 in normal saline prior treatment.

### Experimental Animal and Induction of Diabetes

Male Sprague-Dawley rats weighing 250–300 g (5000*0.1 Gram.1 Digital Scale Balance Lab Analytical Pharmacy Laboratory) purchased from the Animal Research and Service Centre, Universiti Sains Malaysia (USM). The rats were kept in polypropylene cages under standard conditions: 22°C ± 3°C and a 12 h/12 h light/dark cycle. The rats were fed with a commercial diet (Gold Moher, Lipton India, Ltd.) and allowed free access to water ad libitum. All experimental procedures involving animals were conducted in accordance with the guidelines for the care and use of laboratory animals, as approved by the Animal Ethical Committee, Universiti Sains Malaysia Pulau Pinang, Malaysia (USM/Animal Ethics Approval/2013/(89) (479)) and Animal Research and Service Centre (ARASC), USM (Main Campus).

Diabetes was induced by intraperitoneally injecting 55 mg/kg b.w of STZ (Sigma-Aldrich Chemical Co., USA) reconstituted in 0.1 mol/L cold citrate buffer (pH 4.5) after the rats were subjected to fasting overnight. After three days of streptozotocin (STZ) administration, the glucose level measured. For the treatment groups (55 mg/kg of STZ and 500 mg/kg of metformin), the rats weighed and orally administrated using a 16G oral needle. The extraction (500, 250, 125 mg/kg b.w.) freshly prepared in 10 mL of distilled water with 5% of Tween 80 for oral administration, and then the rats weighed every day to determine the volume of sample which needed for administration.

Rats with fasting blood glucose values above 15 mmol/L considered as diabetic rats. Body weight and blood glucose level are recorded before the rats divided into groups. Each group of rats (n = 6) designed and place in one cage by considering the body weight and blood glucose level of all rats for each group and labelled by a permanent marker with a different sign for the following experiment. Control group also labelled by another sign. For each test, we consider six rats in each group: including five diabetic induced groups (30 rats) administration with five crude extracts (chloroform, *n*-hexane, ethyl acetate, methanol, and water), one negative control (*n* = 6) (normal saline administration) and one positive group (metformin administration).

### Blood Sample Collection

The blood were collected from the tail after making a slight cut. A drop of blood was squeezed out and used for the determination of the blood glucose with the glucometer (Accu-check Advantage II clinical glucometer) (Roche Diagnostics Co., USA). In this study, the rats with fasting blood glucose > 15 mmol/L (270 mg/dl) considered as diabetic.

#### Evaluation of the antidiabetic activity of different doses of methanol extract of the Ocimum tenuiflorum leaves

The Sprague Dawley male rats were randomly divided into six groups of six rats in each group and treated (oral administration) for 14 days as follows:

1. Normal Group 1: Control treated with 10 mL/kg normal saline.
2. Diabetic Group 2: Diabetic animals treated with 500 mg/kg metformin.
3. Diabetic Group 3: Treated with 500 mg/kg methanol extract.
4. Diabetic Group 4: Treated with 250 mg/kg methanol extract.
5. Diabetic Group 5: Treated with 125 mg/kg methanol extract.
6. Diabetic Group 6: Diabetic control group treated with 10 mL/kg normal saline.

Fasting blood glucose levels and the change in the body weight of all the rats recorded at 0, 7th and 14th days during the experimental period.

#### Evaluation of the antidiabetic activity of a single dose of different methanol fractions of the Ocimum tenuiflorum leaves

The Sprague Dawley male rats were randomly divided into six groups of six rats in each group and treated (oral administration) for 14 days as follows:

1. Normal Group 1: Control treated with 10 mL/kg normal saline.
2. Diabetic Group 2: Diabetic animals treated with 500 mg/kg metformin.
3. Diabetic Group 3: Treated with 500 mg/kg chloroform extract.
4. Diabetic Group 4: Treated with 500 mg/kg ethyl acetate/butanol extract.
5. Diabetic Group 5: Treated with 500 mg/kg ethanol/water extract.
6. Diabetic Group 6: diabetic control group treated with 10 mL/kg normal saline.

Fasting blood glucose levels and the change in the body weight of all the rats recorded at 0, 7th and 14th days during the experimental period.

#### In vitro α-glucosidase Inhibition Assay

*In vitro*, α-glucosidase inhibition assay was conducted following Apostolidis *et al.* (2006) method with some modification. The α-glucosidase inhibitory activity expressed as inhibition % and calculated as follow:

$$\%\;\text{inhibition}=\frac{\left(\text{average A}405\;\text{control}-\text{average A}405\;\text{extract}\right)}{\text{Average A}405\;\text{control}}\times 100$$

The IC_50_ value was calculated from the dose-response curve by interpolation from the linear regression analysis.

#### In vitro α-amylase Inhibition Assay

*In vitro*, α-amylase inhibition assay was conducted following Apostolidis *et al.* (2006) method with slight modification. The inhibition activity calculated as follows:

$$\%\;\text{inhibition}=\frac{\left(\text{average A}540\;\text{control}-\text{average A}540\;\text{extract}\right)}{\text{Average A}540\;\text{control}}\times 100$$

### Insulin Measurement

The 14 days treated diabetic rats anaesthetised with gas CO_2_, and 3 mL blood was obtained from each rat by the cardiac puncture. The blood samples centrifuged at 3000 rpm for 10 min (Eppendorf, Centrifuge 5403). The plasma was collected and stored at −20°C until measurement. The concentration of insulin in serum assayed in triplicate for each sample using a commercial ELISA kit of rat insulin (Crystal Chem Inc, IL, USA). The insulin concentration was determined via interpolation using the standard curve generated by plotting versus the corresponding concentration of rat insulin standard.

### Statistical Analysis

Values expressed as the standard error of the mean (SEM). A one-way ANOVA employed for the statistical analysis. Graph Pad Prism (version 4) software used for all statistical analyses. *P-values* < 0.05 were significant.

## RESULTS AND DISCUSSION

### Effect of Different Doses of Methanol Extract on Blood Glucose Levels and Body Weight of STZ-Induced Diabetic Rat

**Figure 1:**
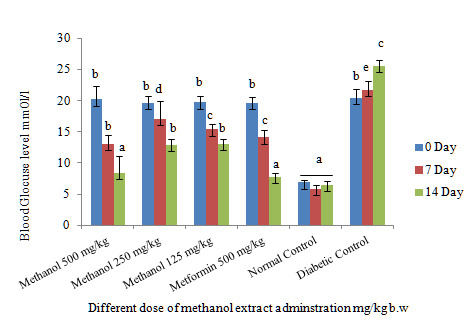
Effect of oral administration of different doses administration of active crude extract and metformin (500 mg/kg) on blood glucose level of STZ-induced diabetic rats. Values are means of *n* = 6 ± SEM, ^a,b,c,d,e^ means on column not sharing the same letter are significantly different (*P* < 0.05).

**Figure 2:**
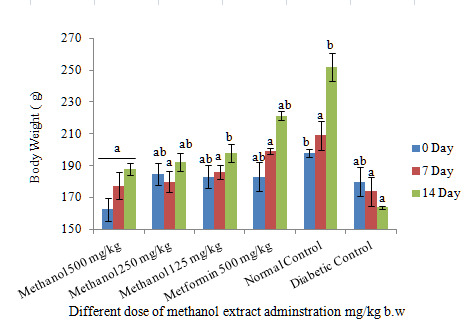
Effect of oral administration of different dose administration of active crude extract and metformin (500 mg/kg) on body weight (g) of STZ-induced diabetic rats. Values are means of *n* = 6 ± SEM, ^a,b,c,d,e^ means on column not sharing the same letter are significantly different (*P* < 0.05).

Fig. 1 shows a decrease in blood glucose levels after the daily administration of different doses of methanol extracts on the day 7 and 14. All doses administered, however, showed a significant decrease in blood glucose levels, but the dose of 500 mg/kg showed the highest reduction in blood glucose levels and indicated nearly the same strength with metformin. The 14 days study of different doses shows that the body weight of all groups gradually increased but no significant difference achieved in comparison to the control group (Fig. 2). However, the 250 mg/kg doses at 14 days showed a significant increase in body weight. Regular increase in rats’ body weight during the 14 days indicates that the various doses of the extract have not led to anorexia, whereas loss of rats’ body weight sometimes results in anorexia. These results are similar to previous reports.

### Effect of Different Doses of Methanolic Fractions on Blood Glucose Levels and Body Weight of STZ-Induced Diabetic Rat

**Figure 3:**
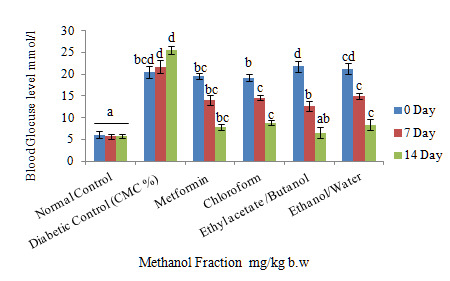
Effect of daily oral administration of fractions, metformin (500 mg/kg) on blood glucose level of STZ-induced diabetic rats. Values are means of *n* = 6 ± SEM, ^a,b,c,d,e^ means on column not sharing the same letter are significantly different (*P* < 0.05).

**Figure 4:**
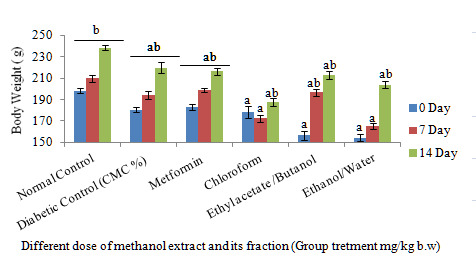
Effect of daily oral administration of fractions, metformin (500 mg/kg) STZinduced diabetic rat. Values are means of *n* = 6 ± SEM, ^a,b,c,d,e^ means on column not sharing the same letter are significantly different (*P* < 0.05).

Fig. 3 shows that the methanol fractions decreased in blood glucose by 500 mg/kg of oral administration in STZ-induced rats during the 14th day. However, the ethyl acetate/butanol fraction of the methanol fractions indicated a high reduction in blood glucose levels up to 14 days compared to the control group and showed nearly the same level of normal blood glucose levels. There were no significant changes in chloroform and ethanol/water fractions as compared to the control group, although blood glucose levels reduced in the 7th and 14th days following treatment. The body weight of the oral administration of fractions during the 14th period showed a gradual increase in body weight but was not significant in comparison with the control group (Fig. 4). The chloroform fraction, however, showed a significant increase in comparison to the control group on the 14th day.

### *In Vitro* α-glucosidase Inhibition Activity

**Figure 5:**
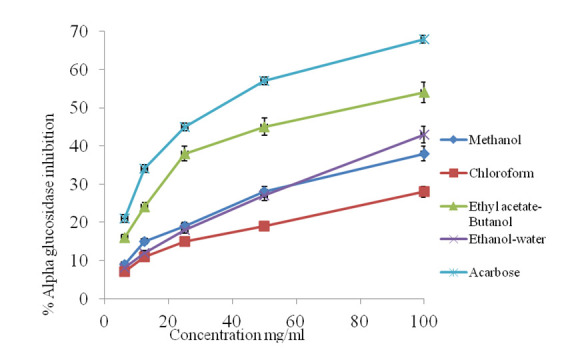
Percentage inhibition of yeast alpha glucosidase enzyme by methanol extract and its fractions of *Ocimum tenuiflorum* L. leaves and reference alpha glucosidase inhibitor, acarbose (values are expressed as mean ± SEM, *n* = 3).

The *in vitro* α-glucosidase inhibitory of methanol extract of *Ocimum tenuiflorum* L. leaves and its fractions exhibited considerable α-glucosidase inhibitory activity (Fig. 5). The percentage inhibition of different dose (mg/mL) concentration of methanol extract showed a concentration-dependent reduction in percentage inhibition. In consequence, the highest level of (100 mg/mL) tested indicated a maximum percentage inhibition of 38%, while the lowest concentration showed the lowest percentage (9%). On the other hands, the chloroform fraction of methanol extract of *Ocimum tenuiflorum* produced a concentration-dependent reduction in percentage inhibition from 28% to 7% against the α-glucosidase activity. Furthermore, the ethyl acetate-butanol and ethanol-water fractions of methanol extract of *Ocimum tenuiflorum* leave produced a concentration-dependent reduction in percentage inhibition between 54% to 16% and 43% to 8% against α-glucosidase activity, respectively. Standard drug (acarbose) at concentrations 100, 50, 25, 12.5 and 6.25 mg/mL produced a concentration-dependent reduction in percentage inhibition against α-glucosidase, which varied between 68% to 21% from the highest to lowest concentration. Also, the *in vitro* α-glucosidase inhibition assay showed that methanol, ethyl acetate-butanol, and ethanol-water fractions were the most potent inhibiting α-glucosidase activity and their IC_50_ were lower than acarbose. While the chloroform extract test requires a higher concentration to inhibit the activity of α-glucosidase (IC_50_: 0.64 ± 0.06) (Table 1).

### *In Vitro* α-amylase Inhibition Activity

**Figure 6:**
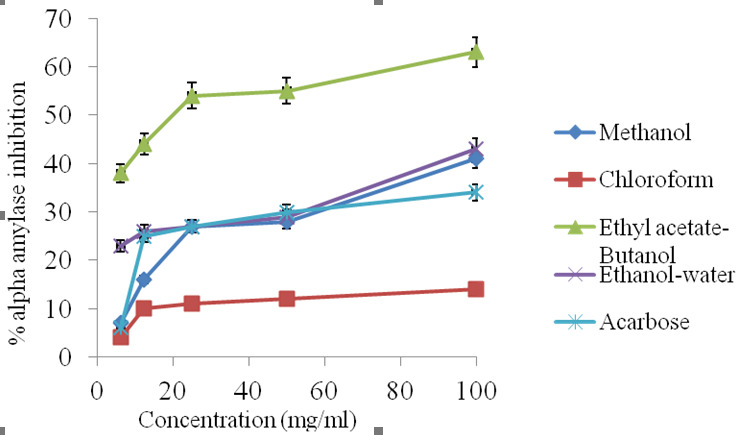
Percentage inhibition of wheat alpha amylase enzyme by methanol extract and its fractions of *Ocimum tenuiflorum* L. leaves and reference alpha amylase inhibitor, acarbose (values are expressed as mean ± SEM, *n* = 3).

The methanol extract of *Ocimum tenuiflorum* leaves produced a concentration-dependent reduction in percentage inhibition against an α-amylase activity as displayed in Fig. 6, which indicated that the highest percentage inhibition was 41% at the concentration of 100 mg/mL. The percentage of inhibition ranged from 41% to 7%. The chloroform fraction also produced a concentration-dependent reduction, with the maximum percentage inhibition around 14% at the highest concentration of 100 mg/mL. The percentage inhibition ranged from 14% to 4% of the highest to lowest level. However, ethyl acetate-butanol fraction showed a concentration-dependent reduction with maximum percentage inhibition at around 63% at the highest concentration and 38% at the lowest concentration.

Furthermore, a concentration-dependent reduction of ethanol-water fraction indicated the reduction around 43% and 23% at the highest and lowest concentration frequently. Acarbose showed a maximum percentage inhibition of around 34% and 6% at the highest and lowest concentration respectively. As results, the *in vitro* α-amylase inhibition assay showed that both ethyl acetate-butanol and ethanol-water are the most potent inhibitors of α-amylase activity with IC50 of 0.59 ± 0.03 and 1.45 ± 0.04, respectively. The IC_50_ of methanol, chloroform was 2.14 ± 0.14 and 2.24 ± 0.09, respectively, which are higher than acarbose 1.54 ± 0.21 (Table 2).

### Insulin Level

**Figure 7:**
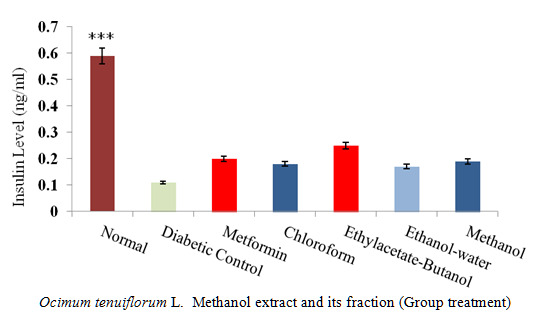
The effect of 14 days daily administration of *Ocimum tenuiflorum* L. methanol extract (ME) 1 g/kg, chloroform, ethyl acetate-butanol, ethanol-water fraction 500 mg/kg on insulin level of streptozotocin-induced diabetic rats. The values are expressed as mean ± SEM (*n* = 3); *** *P* < 0.0001 compared with diabetic control group.

Insulin assay showed that after 14 days of treatment, only insulin levels of normal control were significantly different from diabetic control (*P* < 0.05). In contrast, STZ-induced rat treatment groups (ethyl acetate-butanol fraction, methanol crude extract) clearly showed a higher level than diabetic control but insignificant. The insulin level in treated groups with ethanol-water and chloroform fractions showed almost the same level of control in which the diabetic control was higher, but the results were not significant (Fig. 7).

However, each extract exhibits different solubility properties due to the variety of natural products with different polarities in plant materials. Meanwhile, mainly aqueous plant extracts from traditional herbals are known for their use in disease treatment. Besides, regarding the latest report, most of the researcher has used two or more polar and non-polar solvents consequently for the extraction of a bioactive compound from a medicinal plant (Tédong *et al.* 2007).

Generally, compounds with a low polarity such as some alkaloids, waxes and fatty acids extracted by n-hexane and chloroform (Cowan 1999). Meanwhile, butanol, methanol, and acetate of ethyl are known for the extraction of both medium polarity and certain polar compounds, including some terpenoids, tannins, and flavonoids (Scalbert *et al.* 2005). In contrast, water as a polar solvent showed the derivatives for its extraction activity on highly polar compounds, such as amino acids, carbohydrates, and glycoside (Scalbert *et al*. 2005; Mousavi *et al.* 2018a). However, there is a lack of understanding regarding the mechanisms of action taken. With the inclusion of other possible mechanisms of actions, it occurred hypothesised that absorption might be regulated, which lead to normal glucose homeostasis in diabetic subjects. That is followed by absorption into the blood, where blood-glucose levels stay raised.

These processes regularly happen in the upper portion of the small intestine, results in a notable increase of blood-glucose concentration, particularly in the diabetic patient (Oboh *et al.* 2012). An abnormal high-rise in postprandial blood-glucose concentrations is prevented by the acarbose, due to the α-glucosidase inhibition. Besides, hydrolysation is performed by α-amylase one complex of polysaccharides for the production of oligosaccharides and disaccharides. These two substances are then hydrolysed by α-glucosidase to monosaccharide.

The literature on *Ocimum tenuiflorum* and the previous result has shown that the methanol extract exhibited the maximum reduction in blood glucose level at the dose of 1000 mg/kg. Regarding these results, it was attempted to study the effect of different doses of methanol crude extract in STZ-induced rats for 14 days. The result of dose-dependent oral administration of the different dose of (500, 250, 125 mg/kg) methanolic extract also showed significant reductions of the fasting blood glucose of STZ-induced diabetic rats. The earlier report of ethanolic extract of *Ocimum sanctum* showed the reducing of blood glucose with 130 mg/kg during 10th days treatment compared to the glibenclamide in alloxan-induced diabetic rats by Singh *et al.* (2013). A further study by Gupta *et al.* (2006) indicated the antidiabetic effect of *Ocimum sanctum* seed oil and reduction of the lipid peroxidation and increased glutathione content in the blood.

Nevertheless, two weeks of treatment of *Ocimum sanctum* seed oil in alloxan diabetic rabbit showed no significant hyperglycemic effect (Gupta *et al*. 2006). This finding was in line with another study of aerial part of hydroalcoholic fractions of *Ocimum sanctum* extract on alloxan-induced diabetic rats (Patil *et al.* 2011). However, the present study showed a novel result compared with the previous research since *Ocimum tenuiflorum* L. leave that extracted by a serial solvent extraction process. However, different doses of methanol extract show different potent of antihyperglycemic effect. The body weight of the rats suggested that the administration of extract or fractions did not significantly affect the weight of the rats. Besides that, toxicology assessment of treated rats at the dose of 500, 250, 125 mg/kg indicate no toxic effect and all the rats remain alive until the end of the experiment (Mousavi *et al.* 2018b). The result of body weight during this study did not show any significant changes in different doses of methanol extract administration compared to the control group, due to previous research it is suggested that the oral administered had no deleterious effect on the increase of the body weight of the rats. However, oral administration of 250 mg/kg of methanol extract on the day-7, decreased slightly body weight of the animals. It may be due to abnormalities in glucose metabolism in which energy sullies were not sufficient, causing depletion of fatty acids and proteins to meet energy requirements that could sufficiently be supplied from glucose metabolism. It is also possible that different dose may affect the appetite of rats or involved changes in metabolism efficiency (Trejo-González *et al.* 1996).

The dose of 500 mg/kg of methanol extract showed the highest lowering blood glucose level compared to other doses. Ethyl acetate-butanol fraction indicated the highest lowering in blood glucose level during day-14 treatment. It may be due to the presence of some bioactive compound in this fraction that effectively reduced the glucose metabolism. These results were similar to the previous report of the antidiabetic activity of *Ocimum sanctum* fraction by Patil *et al.* (2011). α-glucosidase and α-amylase suppressors can extend the processes along the intestine, the time taken for carbohydrate absorption is prolonged, and blood glucose concentrations are decreased over the time curve (Hanefeld *et al.* 2004).

A good inhibition on α-glucosidase was exhibited by both ethyl acetate-butanol and ethanol-water fractions (55% and 43%), respectively compared to acarbose at the same range of concentration. This inhibition occurred at a concentration of 100 mg/mL. In contrast, relatively weak enzyme inhibitory activities, with an activity rate of 28%, were produced by chloroform fraction. Based on the observation, it was suggested that most of α-glucosidase inhibited by medium polarity chemical compounds of ethyl acetate-butanol and ethanol-water fractions. In other similar *in vitro* studies, the suppression activity of α-glucosidase in several plant materials had been attributed to the extracts and the presence of polyphenols and flavonoids, along with their glycoside derivatives (Ma *et al.* 2015). Hence, it was reasonable to conclude that the factor for α-glucosidase inhibitory of ethyl acetate-butanol and ethanol-water fractions found in the current study was due to the presence of phenolic compounds and medium polar flavonoids. Moderate inhibitory activities achieved for ethyl acetate-butanol and ethanol-water fractions against α-amylase (63% and 43%) (Fig. 6). The result shows that ethyl acetate-butanol had an IC_50_ value of 0.59 ± 0.03. This concentration was lower than the reference drug acarbose (1.54 ± 0.21 μg/mL) (Fig. 7). Moreover, it can be suggested from these observations that the suppression of α-amylase was due to medium polar extract of plant materials (Tao *et al.* 2013).

Hence, it was possible that the α-amylase inhibitory activity *Ocimum tenuiflorum* extract and its fractions were due to polyphenols, flavonoids and their glycosides. These compounds were known for their solubility in polar and medium polar solvents (Tomsone *et al.* 2012). Additionally, it indicated from the results regarding IC_50_ of α-amylase that ethyl acetate-butanol fractions were possibly more beneficial as postprandial hyperglycemia inhibitor compared to acarbose. It was also shown in the results that the inhibitory action of chloroform and ethanol-water fractions on α-glucosidase were less strong compared to that of acarbose. However, ethyl acetate-butanol fraction and methanol extract displayed stronger effects compared to that of acarbose (Fig. 6).

Furthermore, based on the investigation conducted on insulin level measurement during the 14 days of oral administration of methanol extract and its fraction, it was indicated that a higher level of insulin between other fractions was exhibited by the ethyl acetate-butanol fraction, in comparison to the control group. In regard to the previous study, the most significant hyperglycaemic effect on insulin-dependent and non-dependent diabetes patients exhibited by the herbal treatments of Leguminosae, Lamiaceae, Liliaceae, and Cucurbitaceae (Patel *et al.* 2012). There was a relation between the most significant impact of these plant and their impacts on pancreatic beta cells activities. This was also related to the rise in the enhancement of the sensitivity of the insulin upon the insulin-like activity of plants extracts and the inhibitory impacts against insulinase enzyme, (Bnouham *et al.* 2006). A previous report classified compounds into terpenoids, alkaloids, flavonoids, phenolics, and some other categories, whereby antidiabetic potential could see through the α-glucosidase, α-amylase and insulin level activity (Joseph & Nair 2013). It has also proven that compounds such as chrysin, diosmetin and luteolin are capable for the suppression of α-glucosidase and α-amylase activity. Apart from that, increased insulin secretion will be affected, and insulin sensitivity enhanced by these compounds (Cheng *et al.* 2014).

## CONCLUSION

These results have shown that the active extract of methanol crude and its active fractions (ethyl acetate/butanol) have a significant reduction in glucose because of polyphenolic active ingredients. In brief, form the present outcome, isolation of the active components of *Ocimum tenuiflorum* L. may pave the way to the development of new agents for the treatment of diabetes and its complications.

## Figures and Tables

**Table 1:** IC_50_ of acarbose, methanol crude extract, chloroform, ethyl acetate-butanol and ethanol-water fractions based on α-glucosidase inhibition assay.

Groups	IC_50_ (mg/mL)
Acarbose	0.36 ± 0.21
Methanol	0.05 ± 0.00
Chloroform	0.64 ± 0.06
Ethyl acetate-Butanol	0.05 ± 0.00
Ethanol-Water	0.10 ± 0.00

*Note*: The IC_50_ values were determined from plots of percent inhibition versus log inhibitor concentration and were calculated by non-linear regression analysis from the mean inhibitory values. Acarbose was used as the reference alpha glucosidase inhibitor. All tests were performed in triplicate. Values are expressed as mean ± SEM (n = 3).

**Table 2:** IC_50_ of acarbose, methanol crude extract, chloroform, ethyl acetate-butanol and ethanol-water fractions based on α-amylase inhibition assay.

Groups	IC_50_ (mg/mL)
Acarbose	1.54 ± 0.21
Methanol	2.14 ± 0.14
Chloroform	2.24 ± 0.09
Ethyl acetate-Butanol	0.59 ± 0.03
Ethanol-Water	1.45 ± 0.04

*Note*: The IC_50_ values were determined from plots of percent inhibition versus log inhibitor concentration and were calculated by non-linear regression analysis from the mean inhibitory values. Acarbose was used as the reference alpha amylase inhibitor. All tests were performed in triplicate. Values are expressed as mean ± SEM (*n* = 3).
